# Preliminary Study on the Activity of Phycobiliproteins against *Botrytis cinerea*

**DOI:** 10.3390/md18120600

**Published:** 2020-11-28

**Authors:** Hillary Righini, Ornella Francioso, Michele Di Foggia, Antera Martel Quintana, Roberta Roberti

**Affiliations:** 1 Department of Agricultural and Food Sciences, University of Bologna, Viale G. Fanin, 40, 40127 Bologna, Italy; hillary.righini2@unibo.it (H.R.); ornella.francioso@unibo.it (O.F.); 2 Department of Biomedical and Neuromotor Sciences, University of Bologna, Via Irnerio, 48, 40126 Bologna, Italy; michele.difoggia2@unibo.it; 3 Banco Español de Algas, Universidad de Las Palmas de Gran Canaria, 35214 Telde, Gran Canaria, Spain; amartel@marinebiotechnology.org

**Keywords:** red algae, rhodophyta, cyanobacteria, phycobiliproteins, *Botrytis cinerea*, plant pathogen, gray mold, plant disease control, fungicidal activity, FT-IR, FT-Raman

## Abstract

Phycobiliproteins (PBPs) are proteins of cyanobacteria and some algae such as rhodophytes. They have antimicrobial, antiviral, antitumor, antioxidative, and anti-inflammatory activity at the human level, but there is a lack of knowledge on their antifungal activity against plant pathogens. We studied the activity of PBPs extracted from *Arthrospira* *platensis* and *Hydropuntia* *cornea* against *Botrytis* *cinerea*, one of the most important worldwide plant-pathogenic fungi. PBPs were characterized by using FT-IR and FT-Raman in order to investigate their structures. Their spectra differed in the relative composition in the amide bands, which were particularly strong in *A. platensis*. PBP activity was tested on tomato fruits against gray mold disease, fungal growth, and spore germination at different concentrations (0.3, 0.6, 1.2, 2.4, and 4.8 mg/mL). Both PBPs reduced fruit gray mold disease. A linear dose–response relationship was observed for both PBPs against disease incidence and *H. cornea* against disease severity. Pathogen mycelial growth and spore germination were reduced significantly by both PBPs. In conclusion, PBPs have the potential for being also considered as natural compounds for the control of fungal plant pathogens in sustainable agriculture.

## 1. Introduction

Phycobiliproteins (PBPs) are natural water-soluble proteins present in cyanobacteria and in the chloroplasts of some algae, such as rhodophytes, glaucophytes, and cryptomonas [1,2,3]. They are the main component of light-harvesting complex and can be classified into four types according to their spectral properties: allophycocyanins, phycocyanins, phycoerythrins, and phycoerythrocyanins [4,5]. Phycobiliproteins are of different colors: phycocyanins are blue, allophycocyanins bluish-green, and phycoerythrins are deep red [3]. Moreover, PBPs from different sources could share similar spectral properties, such as the phycocyanins from red algae and those from cyanobacteria. The absorption properties of PBPs are attributed to the presence of open-chain tetrapyrrole chromophores called phycobilins that are capable of capturing sunlight.

Phycobiliproteins have many applications in cosmetics, foods, and medical and diagnostic fields [6,7]. For example, phycocyanins, that are the principal coloring components of *Arthrospira platensis* (syn. *Spirulina platensis*) extract, are food additives falling under EC Regulation No 1333/2008 and were approved as color-safe additives for coloring ingested drugs exempt from certification by the United State Food and Drug Administration in 2013. Many studies have shown antimicrobial, antiviral, antitumor, antioxidative, and anti-inflammatory activity of PBPs at the human level [3,7]. Concerning the antimicrobial activity, PBPs showed efficacy against the human fungus *Candida albicans* and the bacteria *Staphylococcus aureus*, *Streptococcus pyogenes*, *Salmonella typhimurium*, *Pseudomonas aeruginosa*, and *Escherichia coli* [8,9]. Among PBPs, only C-phycocyanin derived from *Spirulina platensis* showed activity against fungal plant pathogens such as *Aspergillus flavus* and species of *Penicillium* and *Rhizopus* [10].

*Botrytis cinerea* Pers. Fr. (teleomorph *Botryotinia fuckeliana* (de Bary) Whetzel) is ranked as the second most important plant-pathogenic fungus [11]. It occurs worldwide and can infect more than 1000 plant species [12]. It causes gray mold, an economically important disease in more than 200 crop species [13]. Favorable conditions, such as high humidity, free moisture on plant surfaces, and moderate temperatures can promote the infection. Besides, the fungus produces an abundant number of spores, infects the leaves, stems, flowers, and fruits of plants, and can survive in a dormant state in the soil [14]. In particular, gray mold is a severe disease on tomato (*Lycopersicon esculentum*), whose production loss can range from 20 to 50% depending on environmental conditions [14]. The pathogen is difficult to control because the infection can remain dormant in the field or greenhouse and develop into fruit decay during post-harvest storage. *Botrytis cinerea* control is mainly based on the use of synthetic fungicides because there are no tomato cultivars resistant to *B. cinerea* commercially available [14]. It is known that the over-use of synthetic pesticides has resulted in risks to human and animal health and the environment [15]. Furthermore, the frequent use of synthetic products acting with a specific mechanism of action has brought pathogen resistance development [16]. Natural compounds for disease management contribute to agricultural sustainability. In Europe, the Directive 2009/128/EC recommends the use of alternative products to synthetic compounds in order to preserve human health and have low environmental impact.

Based on the advice above mentioned, the main objective of this research was to study: (i) the spectral properties of phycobiliproteins (PBPs) extracted from the red alga *Hydropuntia cornea* and the cyanobacterium *Arthrospira platensis*; (ii) the antifungal activity against the gray mold disease on tomato fruits; (iii) the antifungal activity of these PBPs against the growth of *Botrytis cinerea*.

## 2. Results

### 2.1. FT-IR and FT-Raman Spectroscopy Characterization

**Figure 1:**
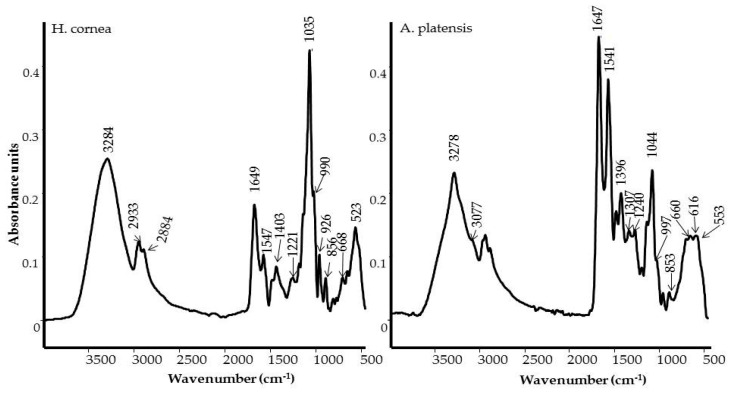
FT-IR spectra of phycobiliproteins (PBPs) from *Hydropuntia cornea* (**left**) and *Arthrospira platensis* (**right**).

**Figure 2:**
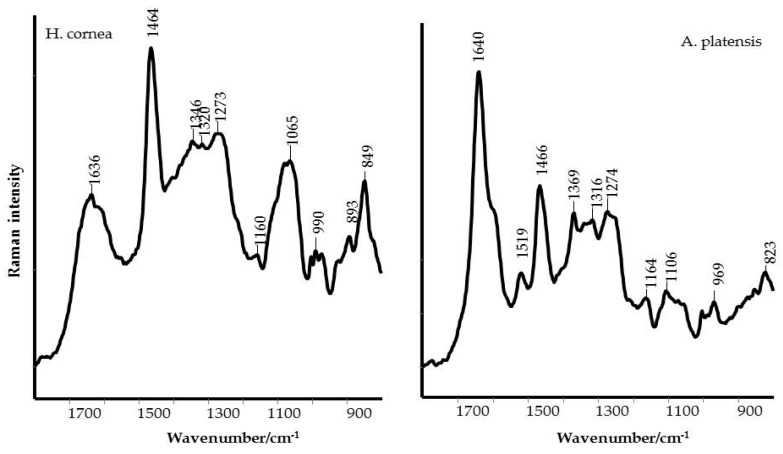
FT-Raman spectra of PBPs from *Hydropuntia cornea* (**left**) and *Arthrospira platensis* (**right**).

In order to characterize and identify the functional groups present in PBPs of *Hydropuntia cornea* and *Arthrospira platensis*, both FT-IR and FT-Raman spectroscopic analysis was performed. The FT-IR profile of PBPs illustrated the specific functional chemical groups, as shown in Figure 1, of proteins [17,18]. The presence of a broad band from 3600 and 3400 cm^−1^ is due to the O–H stretching vibration and water [19]. The peak appearing as a shoulder at 3100–3077 cm^−1^ is assigned to the stretching vibration of the –N–H bond. This band (also known as Amide A) is also typical in secondary amides which are associated with the N–H···O=C type of hydrogen bonding [20]. The bands at around 2960 and 2933–2927 cm^−1^ are attributed to –CH_3_ and –CH_2_ asymmetric stretching vibration, respectively. The peak observed at around 2884–2856 cm^−1^ is assigned to the stretching of –CH_3_, and CH_2_ functional groups. The last two peaks were also detected in the FT-Raman spectra at 2934–2932 and 2901–2889 cm^−1^, as shown in Figure 2. The peak at around 1649–1647 cm^−1^ is referring to the presence of C–O stretching in the peptidic bond (the Amide I band) [20,21]. The peak at around 1547–1541 cm^−1^ is due to C–N stretching coupled to N–H bending in the Amide II band. Additionally, this is representative of the secondary non-cyclic amides [20,21]. The FT-Raman Amide I bands were detected at 1640 and 1636 cm^−1^ for *A. platensis* and *H. cornea*, respectively, as shown in Figure 2. Both spectra showed a shoulder of the Amide I band around 1620–1595 cm^−1^ and attributed to the vibrations of aromatic amino acid such as Tyrosine and Phenylalanine [22]. The Amide II band is usually very weak in the Raman spectrum; therefore, the band appearing at 1518 cm^−1^ in the spectrum of *A. platensis* should be attributed to carotenoid pigments, together with the bands at 1160 and 1000 cm^−1^ [23]. PBP spectra differed in the relative intensity of the amide bands, which were particularly stronger in *A. platensis*, as shown in Figure 1. The position and shape of the Amide I band did not differ in both PBPs, indicating an overall α-helix structure adopted by PBPs. The FT-Raman Amide I bands confirmed both findings, while the highest FWHM (full width at half maximum) indicates that proteins in *H. cornea* have a higher content of random structures [24].

The band at 1403 cm^−1^ in the FT-IR spectrum is assigned to –COO^−^ symmetric stretching, symmetric deformation of –CH_3_, and CH_2_ functional groups, and the deformation of the –OH group. The other bands observed between 1300 and 1221 cm^−1^ are attributed to the vibration involving C–N stretching and N–H bending in Amide III [20]. While the carboxylate vibration was not detected in the Raman spectrum, the position of the peaks in the Amide III region further supported that PBPs adopted an overall α-helix/random structure; the peak at 1273 cm^−1^ is typical of an α-helix structure, while the peak (shoulder in *H. cornea*) at 1250 cm^−1^ indicates a random structure [24]. Secondary amides are also characterized, in the FT-IR spectrum, by other bands at around 700 cm^−1^ due to the N–H out of plane deformation (Amide V), at around 625 cm^−1^ to O=C–N bending (Amide IV), and 600–535 cm^−1^ assigned to C=O out of plane bending (Amide VI). Moreover, the band at around 660 cm^−1^ may be assigned to the –C≡C–H stretching vibrations in alkynes [20]. The presence of a band at around 1044–1035 cm^−1^ is typical of a C–O–H stretching bond in primary and secondary alcohols, with a C–C and C–O–C stretching motion [20]. The bands at 926 cm^−1^ and 856–853 cm^−1^ can be assigned to C–H out of plane deformation, respectively.

In *H. cornea*, the band at 1035 cm^−1^ was the most prominent one in the FT-IR spectrum, as shown in Figure 1. However, a mineral compound such as silicate could contribute to the intensification of this band [18]. FT-Raman spectrum of *H. cornea*, as shown in Figure 2, confirmed the presence of silicates with two intense bands at 1065 (very large band mixed with C–O–H stretching bands of alcohols) and 848 cm^−1^ [25].

**Figure 3:**
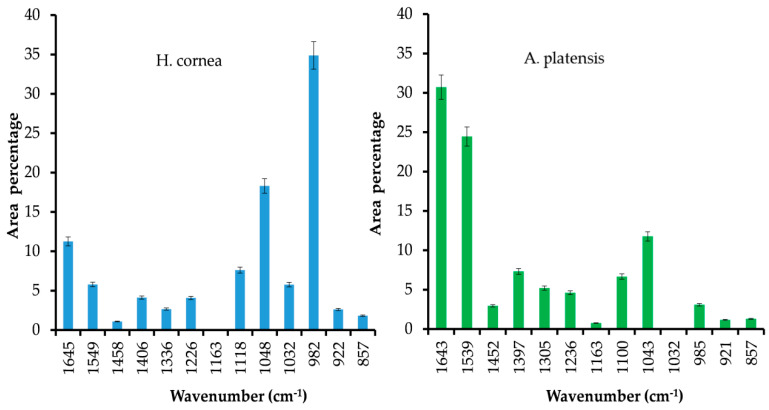
Area percentage of more significant peaks of PBPs from *Hydropuntia cornea* and (**left**) and *Arthrospira platensis* (**right**) in the region between 1800 and 800 cm^−1^. The peaks were processed using a Gaussian curve peak fitting. Bars correspond to standard error.

Semiquantitative estimation of the contribution of different bands of the FT-IR spectra in the region between 1800 and 800 cm^−1^ was obtained using Gaussian curve peak fitting, as shown in Figure 3. Amide I and Amide II accounted for 11 and 6% in *H. cornea* and 31 and 24% in *A. platensis*, respectively. The area percentage of the peaks (~1458 and ~1408 cm^−1^) related to CH in aliphatic compounds accounted for 1 and 4% in *H. cornea* and 3 and 7% in *A. platensis*, respectively. Amide III accounted for 2 and 4% (1336 and 1226 cm^−1^) in *H. cornea* and 5.2 and 4.6% (1305 and 1226 cm^−1^) in *A. platensis*, respectively. The area percentages of functional groups attributed to primary and secondary alcohols accounted for 8, 18, and 6% (1118, 1048, and 1032 cm^−1^) in *H. cornea*, respectively, and accounted for 7 and 12% (1100, 1043 cm^−1^) in *A. platensis*, respectively. The area percentage of C–C stretching vibration at around 985 cm^−1^ accounted for 35% in *H. cornea* and 3% in *A. platensis*, respectively. Usually, this type of bond is not intense, but the oxygen bound to the carbon atoms polarizes the bond with a consequently increased peak intensity [20].

### 2.2. Effect of PBPs against B. cinerea on Tomato Fruits

**Figure 4:**
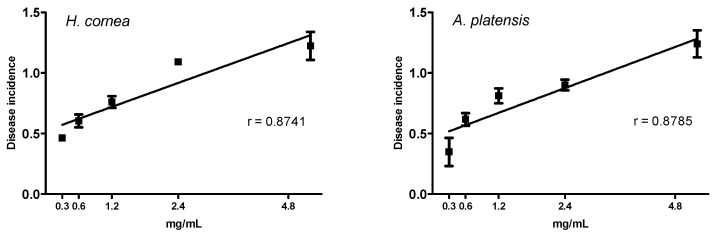
Linear regression of the activity of different doses of PBPs from *Hydropuntia cornea* (y = 0.523094 + 0.164224x, *p* < 0.0001) and *Arthrospira platensis* (y = 0.46725 + 0.169844x, *p* < 0.0001) against the *Botrytis cinerea* disease incidence of tomato fruits. Showed data are arcsine transformed. Each value (*n* = 4) is a mean ± standard error (SE).

**Figure 5:**
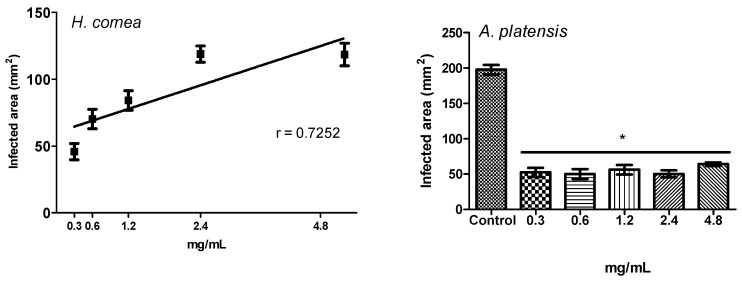
Linear regression of the activity of different doses of PBPs from *Hydropuntia cornea* (y = 60.2313 + 14.7036x, *p* < 0.0001) against the *Botrytis cinerea* disease severity on tomato fruits (**left**) and activity of different doses of PBPs from *Arthrospira platensis* against disease severity (**right**). Each value (*n* = 4) is a mean ± SE. For *A. platensis*, F_(5,18)_ = 95.94 (*p* < 0.05), the asterisk indicates significant difference of treated fruits against untreated according to Tuckey’s multiple comparison test (*p* < 0.05).

The antifungal activity of different concentrations of PBPs from *H. cornea* and *A. platensis* against the *B. cinerea* disease is reported in Figure 4 and Figure 5 for both disease incidence and disease severity, respectively. Concerning the disease incidence, a dose–response relationship described by a linear model was observed for both PBPs, as shown in Figure 4. The two PBPs showed the same effect against disease incidence as demonstrated by the statistical identity of the two slopes, F_(1,36)_ = 0.0288, P = 0.8662. All PBP doses significantly reduced the disease incidence with respect to the control (untreated) by 81.7, 65.4, 47.7, 26.8, and 10.5% at 0.3, 0.6, 1.2, 2.4, and 4.8 mg/mL, respectively.

**Figure 6:**
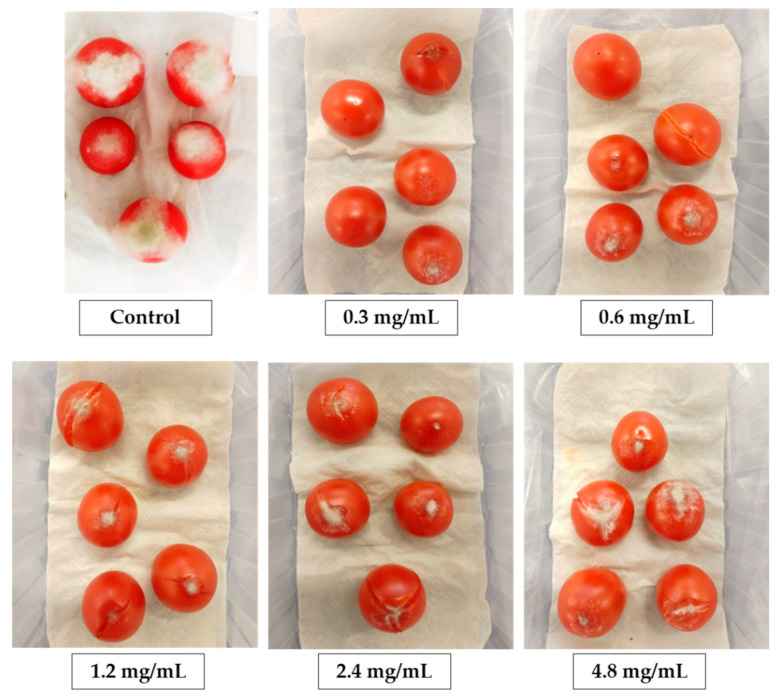
Effect of treatment with PBPs from *Hydropuntia cornea* at different concentrations on gray mold of tomato fruits.

About disease severity, a dose–response relationship described by a linear model was observed for *H. cornea* PBPs, as shown in Figure 5 and Figure 6, and all PBP doses significantly reduced the infected area with respect to the control (untreated) by 78.1, 66.5, 59.8, 43.2, and 43.4% at 0.3, 0.6, 1.2, 2.4, and 4.8 mg/mL, respectively. For *A. platensis* PBPs, no dose–response relationship was observed; however, all doses significantly similarly reduced disease severity by an average of 72.4%.

### 2.3. Effect of PBPs on Fungal Growth

**Figure 7:**
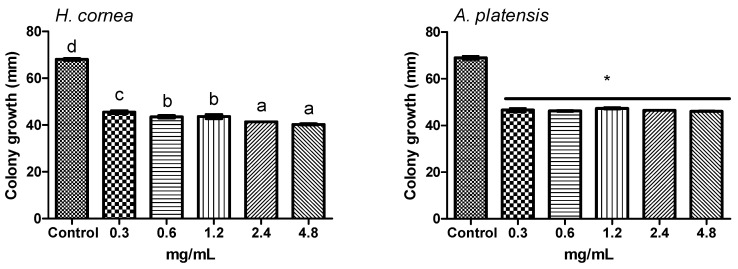
Effect of treatment of *Botrytis cinerea* colony portions with different concentrations of PBPs from *Hydropuntia cornea* and *Arthrospira platensis* on colony growth at 3 days after treatment. Columns are means values (*n* = 4) ± SE; F_(5,18)_ = 399.23 (*H. cornea*), F_(5,18)_ = 248.77 (*A. platensis*). Different letters and the asterisk indicate significant differences according to Tuckey’s multiple comparison test (*p* < 0.05).

The effect of different concentrations of *H. cornea* and *A. platensis* PBPs on *B. cinerea* colony growth at 3 days after treatment of fungal portions is reported in Figure 7. No dose–response relationship between each PBP treatment and colony growth was observed. One-way ANOVA indicated that *H. cornea* PBPs reduced pathogen growth at all doses with respect to the control (0.0 mg/mL) by 33.0, 36.0, 35.8, 39.1, and 40.7% at 0.3, 0.6, 1.2, 2.4, and 4.8 mg/mL, respectively. The most significant effective doses in reducing colony growth were 2.4 and 4.8 mg/mL. The different concentrations of *A. platensis* PBPs significantly reduced colony growth in the same way by an average of 32.5% with respect to the control. 

**Figure 8:**
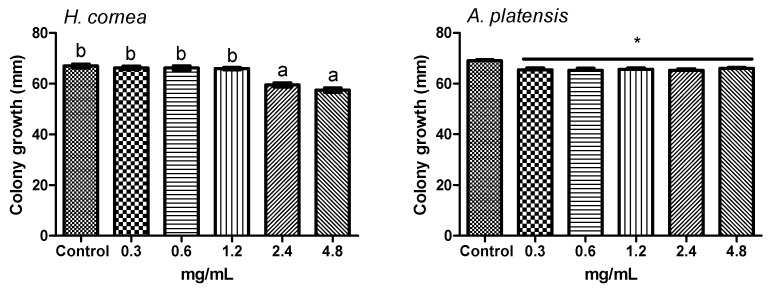
Growth of *Botrytis cinerea* colonies on potato dextrose agar (PDA) amended with different concentrations of PBPs from *Hydropuntia cornea* and *Arthrospira platensis* at 3 days after inoculation. Columns are means values (*n* = 4) ± SE; F_(5,18)_ = 29.58 (*H. cornea*), F_(5,18)_ = 5.75 (*A. platensis*). Different letters and the asterisk indicate significant differences according to Tuckey’s multiple comparison test (*p* < 0.05).

Figure 8 shows the colony growth of *B. cinerea* on potato dextrose agar (PDA) amended with different concentrations of PBPs at 3 days after colony inoculation. *Hydropuntia cornea* PBPs significantly reduced the pathogen growth at 2.4 and 4.8 mg/mL in a similar way by an average of 12.7% with respect to the control (0.0 mg/mL), while *A. platensis* PBPs significantly reduced the growth by an average of 5.0%, regardless of the dose.

### 2.4. Effect of PBPs on Fungal Colony Forming Units (CFUs)

**Figure 9:**
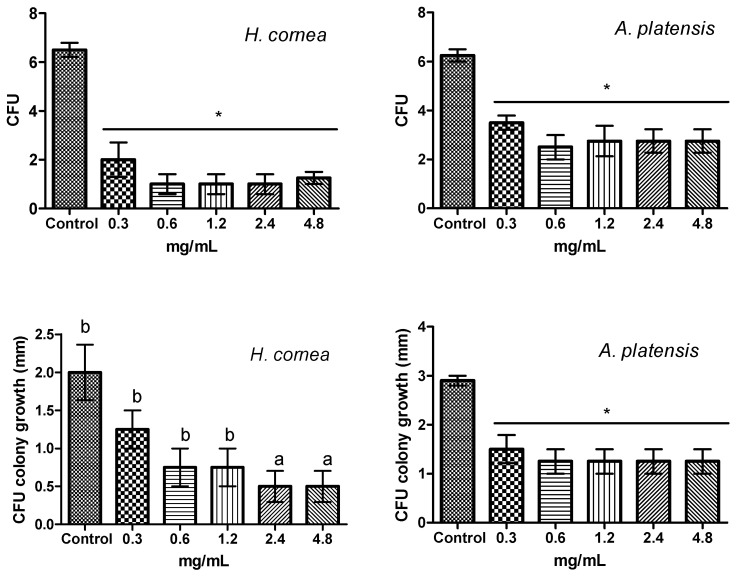
Effect of PBPs from *Hydropuntia cornea* and *Arthrospira platensis* on colony forming units (CFUs), and on CFU colony growth of *Botrytis cinerea* at 2 days after spore treatment. Columns are mean values (*n* = 4) ± SE. For CFUs, F_(5,18)_ = 24.84 (*H. cornea*), F_(5,18)_ = 9.80 (*A. platensis*). For CFU colony growth, F_(5,18)_ = 20.10 (*H. cornea*), F_(5,18)_ = 7.63 (*A. platensis*). Different letters and the asterisk indicate significant differences according to Tuckey’s multiple comparison test (*p* < 0.05).

Figure 9 reports the effect of the different concentrations of PBPs from *H. cornea* and *A. platensis* against *B. cinerea* colony forming units (CFUs) and on CFU colony growth at 2 days after spore treatment. Both PBPs significantly reduced CFUs regardless of the dose. *Hydropuntia cornea* PBPs reduced CFUs by an average of 80.8% (Figure S1a), while those of *A platensis* by 54.4% with respect to the control.

For CFUs fungal colony growth, PBPs of *H. cornea* were significantly effective at 2.4 and 4.8 mg/mL in a similar way by an average of 84.6% towards the control. *Arthrospira platensis* PBPs reduced the colony growth by an average of 55.2% with respect to the control regardless of the dose.

Microscopical observations of *B. cinerea* spores, which were treated with PBSs, revealed that spores germinated poorly by producing short germ tubes and that no hyphal branches occurred. On the contrary, conspicuous hyphal ramifications originated from untreated spore. Supplementary materials S1b shows the effect of PBPs from *A. platensis* on spore germination compared to the control.

## 3. Discussion

Phycobiliproteins of red algae and cyanobacteria are valuable products with several commercial applications and are widely studied for their antimicrobial, antiviral, anticancer, and antioxidant properties at the human level [8,9,26,27,28].

This study was carried out to investigate the potential antifungal activity of PBPs extracted from the red alga *Hydropuntia cornea* and the cyanobacterium *Arthrospira platensis* against the fungal plant pathogen *Botrytis cinerea*. The experiments were divided into two groups.

In the first one, the antifungal activity was assessed on tomato fruits artificially infected with *B. cinerea* after PBP treatment and in the second group the antifungal activity of PBPs was assayed on both colony growth and spore germination of the pathogen. As far as we know, there is no available literature on the use of PBPs against fungal plant pathogens, while several studies have been carried out in the medical field. Our findings showed that the antifungal activity of PBPs depends on their chemical structure and concentration and that they may interfere with *B. cinerea* development. The spectroscopic analyses of PBPs, using IR and Raman techniques, pointed out some important differences between *H. cornea* and *A. platensis* in the content and secondary structure of the proteins, and on the presence of polar groups that could influence the overall polarity of the PBPs. In more detail, *A. platensis* showed a higher relative intensity of the bands related to the vibrations of the peptide bond compared to *H. cornea* with both spectroscopic techniques. Moreover, the semiquantitative analysis using curve peak fitting indicated that the contribution of protein bands (areas of Amide I, Amide II, and Amide III bands) in the 1800–800 cm^−1^ IR spectral range accounted for 65% in *A. platensis* and 23% in *H. cornea*, as shown in Figure 3. In this last species, very intense bands in the IR spectrum were detected at 1048 and 982 cm^−1^ and attributed to mixed vibrations involving C–C, C–O, and C–OH bonds of alcohols.

Taking into account the secondary structure of proteins, the increased FWHM (full width at half maximum) of the Raman Amide I band of *H. cornea*, a higher contribution of random coil structures can be argued [24], while *A. platensis* showed a typical α-helix structure, as shown in Figure 2. This secondary structure difference between the two extracts can be responsible for the mostly dose–response independent antifungal activity displayed by *A. platensis*, probably because the tested doses were too high. Transmembrane proteins are more abundant in the α-helix class [29]; therefore, a higher interaction with the fungal cell wall can be hypothesized with a consequent perturbation of cell activity. On the other hand, for *H. cornea*, the antifungal effect was mostly linear dose–response dependent. This effect is enhanced by the highest hydrophilicity of the protein in which the bands attributed to polar functional groups (i.e., C–OH and C–O typical of alcohols) have a higher contribution, as shown in Figure 3. We may speculate that both PBPs, having distinct hydrophilic and hydrophobic regions, may interact differently with *B. cinerea* hydrophobins. The latter proteins can assemble spontaneously into amphipathic monolayers at hydrophobic–hydrophilic interfaces by covering the fungal surface. They are considered an important pathogenesis factor in the interaction between the fungus and the host [30]. This interaction can be involved in the PBPs’ effectiveness against the gray mold disease we obtained on tomato fruits.

The PBPs’ antifungal effect against *B. cinerea* could also be related to an antioxidant activity. Indeed, several studies reported that PBPs are a natural free radical scavenger [3,7]. Oxidative reactions and reactive oxygen species (ROS) are involved in various human diseases such as atherosclerosis, cancer, ototoxicity, and diabetes and Alzheimer’s [1,2,9,31,32]. The PBP phycoerythrin extracted from *Michrochaete* sp. showed a scavenging effect for DPPH similar to that of ascorbic acid resulting as a potent free radical scavenger [9]. Pre-treatment with phycocyanin from the cyanobacterium *Limnothrix* sp. inhibited apoptosis and protected mitochondrial function by preventing reactive oxygen species (ROS) accumulation in cisplatin-treated HEI-OC1 cells, a mouse auditory cell line [1]. Another study showed that in HepG2 cells, a human liver cancer cell line, treated with PBP reduced hydrogen peroxide-mediated oxidative stress and restored the expression of the enzyme superoxide dismutase, an important antioxidant defense against oxidative stress in the human body [2]. These authors also showed that treatment with phycobiliproteins upregulated the level of the phosphorylated nuclear factor erythroid-derived 2-like 2, a basic leucine zipper protein that regulates the expression of antioxidant proteins that protect against oxidative damage triggered by injury and inflammation.

The antifungal activity of *H. cornea* and *A. platensis* PBPs was noticeable both against mycelial growth and spore germination detected as CFUs. A clear colony growth inhibition was observed when fungal colony portions were treated by immersion in a PBP solution, this allowing a complete contact of the mycelium with the proteins. Microscopic observations revealed that PBPs hampered the fungus spore causing a reduction of germination and of hyphal development (Figure S1b). Spores are the dispersal means of *B. cinerea* that are the origin of many secondary infections on plants. The antifungal activity of PBPs on fungal spore germination can have a role in disease control, because the life cycle of the pathogen may be interrupted. Other kinds of proteins, such as those derived from *Aspergillus giganteus*, showed antifungal activity against *B. cinerea* by reducing hyphal elongation and spore germ tube length, and by completely inhibiting spore germination at the highest protein concentration [33]. Indeed, proteins and peptides with antifungal activity against phytopathogenic fungi have been demonstrated to affect fungal membranes causing membrane permeabilization, formation of pores, and alteration in morphology of hyphae, eventually leading to fungal cell death [33,34,35].

Antifungal activity of PBPs was reported for the B-phycoerythrin and C-phycocyanin of the cyanobacteria *Synechocystis* sp. and *Arthrospira fusiformis* and of the red alga *Porphyridium aerugineum* against spore growth of the human fungal pathogen *Candida albicans* [8]. These authors showed that PBPs display different effects depending on algal and cyanobacteria species. Phycobiliproteins also exerted antibacterial activity against *Staphylococcus aureus*, *Streptococcus pyogenes* and *Salmonella typhimurium*, *Pseudomonas aeruginosa*, and *Escherichia coli* [8,9].

In the present study, the pre-treatment with PBPs of tomato fruits before the *B. cinerea* challenge reduced both the disease incidence and the severity at all concentrations. We can assume that reduction of *B. cinerea* symptoms on tomato fruits is due to the direct effect of phycobiliproteins against the pathogen, as noted in vitro and above reported. However, we cannot exclude a combination of the direct effect against the pathogen with an induction of fruit defense responses, activated by the PBPs.

It should be pointed out that PBPs from *A. platensis* and *H. cornea* showed a typical biostimulant behavior, because they both reduced fruit disease incidence and only those from *H. cornea* reduced disease severity more at lower concentrations than at the highest ones. Biostimulants are substances, or microorganisms, that applied in low quantities can promote plants’ nutrition efficiency, abiotic stress tolerance, and crop quality and yield [36]. Among biostimulant substances, algal extracts are included.

In conclusion, PBPs have the potential for being also considered as natural compounds for the control of fungal plant pathogens. Since they are substances already used in human consumption, they could be considered in the list of the basic substances, a newly effective category of the plant protection products provided by the EC Regulation No 1107/2009. At present, the exact mechanism by which PBPs from *A. platensis* and *H. cornea* exert their direct antifungal activity against *B. cinerea* is, however, unknown and further investigations need to clarify the potential role in plant/fruit resistance induction.

## 4. Materials and Methods 

### 4.1. Hydropuntia cornea and Arthrospira platensis Culture, PBP Extraction and Purification

*Hydropuntia cornea* (J. Agardh) M.J. Wynne 1989 (formerly *Gracilaria cornea*) was cultivated outdoors at the greenhouse facilities of the Spanish Bank of Algae (BEA, Taliarte, Gran Canaria, Spain), in a set of semicircular fiberglass aerated tanks (750 L) at an optimal density of 6 g/L. N-NH_4_^+^ (200 µM) and P-PO_4_^3−^ (20 µM) enriched seawater was continuously pumped to the tanks at turnover rates of 4 vol/d. Relative growth rates µ (% d^−1^) were calculated according to the following equation [37]: µ = 100 ln (W_t_/W_0_)/t [37], where W_0_ = initial fresh weight, W_t_ = final fresh weight, and t = time in days reaching values between 5 and 9% d^−1^ during the experimental period.

*Arthrospira platensis* (M. Gomont, 1892) BEA 0007B, obtained from BEA Culture Collection of Microalgae and Cyanobacteria, was cultivated outdoors at the BEA greenhouse facilities in a polypropylene open raceway pond (10 m^2^; 1200 L). The open channel raceway was 6 m long, had a 1.3 m width, and 0.25 m depth. A single, seven-bladed paddle wheel with the rotating speed of 6 rpm was used. The culture media composition contained 95.24 mM NaHCO_3_, 584.10 mM NaCl, 19.78 mM KNO_3_, 0.19 mM MgSO_4_·7H_2_O, 0.70 mM NH_4_H_2_PO_4_, 1.25 mM Na_2_ EDTA·2H_2_O, 0.25 mM Urea, and 0.02 mM FeSO_4_·7H_2_O. Growth was monitored daily by measuring optical density at 680 nm in a spectrophotometer. 

For both outdoor cultures, the biomass was weekly harvested by filtration, washed with distilled water, and dehydrated using a B. Master Plus dehydrator (Tauro Essicatori). Maximum irradiance levels reached 1800 µmol photon m^−2^ s^−1^, and the water temperature fluctuated between 20 and 24 °C.

Fifteen grams of the dry thallus of *H. cornea* and 7.5 g of lyophilized biomass of *A. platensis* were suspended in 100 mL of 0.2 M phosphate buffer, pH 7. The suspensions were stirred for 4 h at room temperature in the dark and then centrifuged for 20 min at 13 °C, 5000 rpm. The PBPs were desalted and separated from algal and cyanobacterial cell residues by using Amicon^®^ Ultra-4 (Millipore Corporation, Burlington, MA, USA) centrifugal filtering devices, and then filtered with a GV Millex^®^ Syringe Filter Unit (pore diameter 0.45 μm, Millipore Corporation, USA). After purification, PBPs were lyophilized and stored at −80 °C until use.

### 4.2. FT-IR and FT-Raman Spectroscopy of PBPs

The FT-IR spectra of PBPs were collected using an Alpha FT-IR instrument (Bruker Optics, Ettlingen, Germany) provided with an ATR (attenuated total reflection) diamond crystal sampling device. The spectra were measured between 4000 and 400 cm^−1^, with a spectral resolution at 4 cm^−1^, and 64 scans. A background spectrum against air was recorded under the same conditions before each series of measurements. For the measures, less than 1 mg of sample was used. Offset normalization was conducted to adjust the baseline and move the spectrum intensities so that the minimum absorbance value was 0. The peak fitting was performed between 1800 and 800 cm^−1^ using Gaussian functions. All spectra were processed using the Grams/386 spectroscopic software (version 6.00, Galactic Industries Corporation, Salem, NH, USA). The best-fit parameters were calculated by the values of reduced chi-square (χ^2^), the determination coefficients, R^2^, (ranging from 0.996 to 0.993) and the standard error, SE, from 0.004 to 0.002. All data were shown in percentage area. Raman spectra were recorded by using a Bruker MultiRam FT-Raman spectrometer equipped with a cooled Ge-diode detector. The excitation source was an Nd^3+^-YAG laser (1064 nm) in the backscattering (180°) configuration. The focused laser beam diameter was about 100 µm, the spectral resolution 4 cm^−1^, and the laser power at the sample about 50 mW. Each Raman spectrum was the average of 5000 spectra.

### 4.3. Pathogen Culture

The fungal pathogen *B. cinerea* 06 belonging to the DISTAL collection was cultured on potato dextrose agar (PDA, 4%, Difco, Laboratories, Detroit, MI, USA) at 25 °C for 14  days. Fungus pathogenicity was verified through tomato fruit inoculation with a spore suspension. Spore suspension of *B. cinerea* was prepared in sterile distilled water by removing the spores from the sporulating edges of a 14 day old colony with a bacteriological loop. The suspension was filtered through a sterile cheesecloth (100 μm pore size) and adjusted to a final concentration of 10^6^ spores/mL using a hemocytometer. Tomato fruits (Pachino Cherry, Moncada, Soc. Agr. Coop. O.P., Sicily, Italy) were surface-disinfected by 2.5% (*v/v*) sodium hypochlorite for 3 min, rinsed twice with sterile distilled water, and then air-dried under a sterile flow cabinet. Fruits were placed in a plastic container on sterile wet paper, wounded (5 mm deep) with a sterile needle in the eye cavity, and each wound was inoculated with 10 µL of the spore suspension. For the control, 10 µL of sterile distilled water was used. Inoculated and control fruits were enclosed in a plastic bag and then incubated at 24 °C in a growth chamber until symptom appearance.

### 4.4. Effect of PBPs against Gray Mold on Tomato Fruits

Healthy tomato fruits (*Solanum lycopersicum* L. cv. ciliegia F1) were harvested from the greenhouse of the Department of Agricultural and Food Sciences, Bologna, at the ripened stage and immediately transported to the laboratory. Fruits with uniform size and free of defects were selected, washed, and surface sterilized by immersing them in a 2.5% (*v*/*v*) sodium hypochlorite solution for 3 min, rinsed with sterile distilled water two times, and then air-dried under a sterile flow cabinet. Five sterilized fruits were placed in plastic containers on sterile wet paper and wounded (5 mm deep) on the blossom end with a sterile needle [38]. For the treatment, 10 μL of each PBP concentration was pipetted onto each wound site and, after 30 min, 10 μL of spore suspension (10^6^ spores/mL) was inoculated. Two controls were considered; one consisted of fruits treated with 20 μL sterile distilled water (negative control) and one of fruits treated with 10 μL of sterile distilled water and then inoculated with 10 μL of *B. cinerea* spore suspension (positive control). Four replicates (*n* = 4) were considered for each treatment and the controls. The containers were enclosed in a plastic bag in order to maintain high humidity and incubated at 24 °C in a growth chamber with a 12 h/12 h day/night photoperiod. After 10 days, gray mold symptoms were evaluated as disease incidence and disease severity. For the disease index, diseased fruits were counted on the total of inoculated fruits. For disease severity, images of diseased fruits were taken, and the diseased area (mm^2^) was measured with Image Processing and Analysis (National Institute of Health).

### 4.5. Effect of PBPs on Botrytis cinerea Colony Growth

The effect of PBP on fungal colony growth was performed by submerged colony and agar dilution technique [39,40] methods with modifications. Different PBP concentrations were used: 0.3, 0.6, 1.2, 2.4, and 4.8 mg/mL. For the submerged colony technique, portions of 6 mm diameter were cut from the 14-day-old colony and treated by immersion in a test tube containing 1 mL aliquot of each PBP concentration for 6 h. Sterile distilled water was used as control. Colony portions were then placed on a PDA medium in a Petri dish and incubated at 24 °C for 3 days in the dark. For the agar dilution method, autoclaved PDA medium was cooled until 40 °C and added with an aliquot of PBP solution to have the final concentrations reported above. Sterile distilled water was used as the control. The medium was poured into Petri dishes (9 cm diameter) and inoculated with one colony portion (6 mm diameter) in the center of the dish as it solidified. Dishes were incubated at 24 °C for 3 days in the dark. In both experiments, colony growth was measured daily in two directions, along two mutually perpendicular diameters. Four dishes were considered for each treatment and water control. The experiment was repeated twice.

### 4.6. Effect of PBPs on Botrytis cinerea Colony Forming Units (CFUs)

The effect of PBPs on CFUs was performed by preparing in test tubes a mixture of each PBP concentration with *B. cinerea* spore suspension 5 × 10^2^ spores/mL. Sterile distilled water was considered as a control. After 4 h of incubation, 20 µL from each mixture was gently spread on the surface of a PDA medium in a Petri dish. Four dishes were considered for each PBP concentration and the control. Dishes were incubated at 24 °C in the dark, and after 48 h, the CFU number was counted, and the diameter of the colonies derived from CFUs was measured along two mutually perpendicular diameters in each dish. The experiment was repeated twice.

Microscopical observation of *B. cinerea* spore germination was carried out after 24 h from the treatment. From each test tube containing the same mixture prepared for CFU determination, four drops of 50 µL volume were put on microscope glass slides and observed on an optical microscope (Nikon Eclipse TE2000 E Microscope, Nikon Instruments Europe BV, Amsterdam, The Netherlands).

### 4.7. Statistical Analysis

A linear regression was performed to test the dose response of both PBPs at different concentrations towards disease incidence and disease severity of tomato fruits. The percentage of disease incidence data were arcsine transformed before linear regression analysis (*p* < 0.05). Transformed data were used in graphs. An ANOVA (*p* < 0.05), followed by Tuckey’s test (*p* < 0.05), was performed to process all results without any dose–response relationship. All analyses were performed with GraphPad Prism software, version 5.01.

## Supplementary Materials

The following are available online at https://www.mdpi.com/1660-3397/18/12/600/s1, Figure S1a: Effect of PBPs from *Hydropuntia cornea* at 0.3 mg/mL on *Botrytis cinerea* colony forming units (a) compared to the control (b), 3 days after treatment, Figure S1b: Effect of PBPs from *Arthrospira platensis* at 0.6 mg/mL on *Botrytis cinerea* spore germination (c, d, e, f) compared to hyphal development from untreated spore (a, b), 24 hours after treatment, Charts of Figure 4, Figure 5, Figure 7, Figure 8 and Figure 9.
