# Supplementary material for: Preliminary Study on the Activity of Phycobiliproteins against Botrytis cinerea
Source: Mar Drugs. 2020 Nov 28;18(12):600. doi: 10.3390/md18120600 (PMC7759837; doi:10.3390/md18120600)
Supplement: Supplementary file 1 [file marinedrugs-18-00600-s001.pdf]

**Supplementary material: S1a, S1b.**

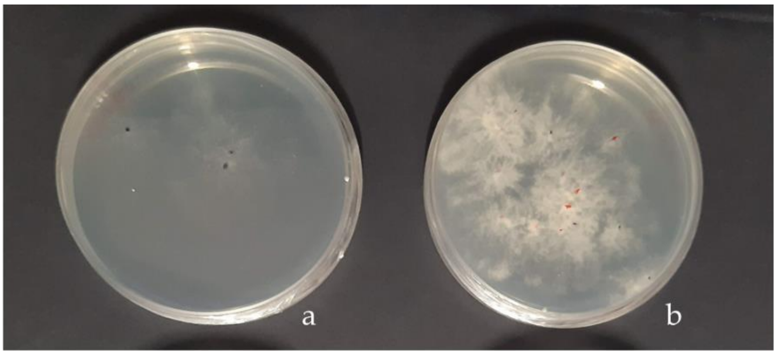


Figure S1a. Effect of PBPs from *Hydropuntia cornea* at 0.3 mg/mL on *Botrytis cinerea* colony forming units (a) compared to the control (b), 3 days after treatment.

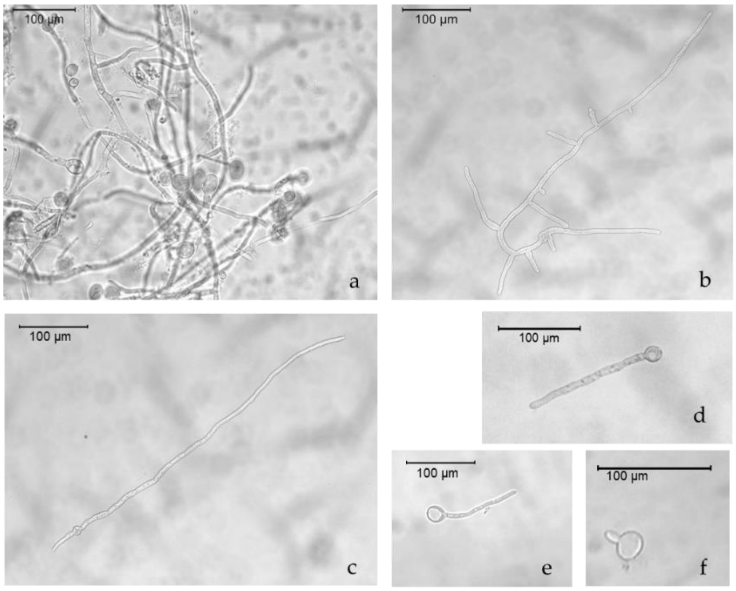


Figure S1b. Effect of PBPs from *Arthrospira platensis* at 0.6 mg/mL on *Botrytis cinerea* spore germination (c, d, e, f) compared to hyphal development from untreated spore (a, b), 24 hours after treatment.

**Supplementary charts**

Table of Fig. 4 – Activity of different doses of PBPs from *Hydropuntia cornea* and *Arthrospira platensis* against the *Botrytis cinerea* disease incidence of tomato fruits. Data were arcsin transformed. Each value ($n$ = 4) is a mean ± SE.

| Treatment mg/mL | *H. cornea* | *A. platensis* |
|---|---|---|
| 0.3 | 0.46 ± 0.00 | 0.35 ± 0.12 |
| 0.6 | 0.60 ± 0.05 | 0.62 ± 0.05 |
| 1.2 | 0.76 ± 0.05 | 0.81 ± 0.06 |
| 2.4 | 1.09 ± 0.01 | 0.90 ± 0.04 |
| 4.8 | 1.22 ± 0.12 | 1.24 ± 0.11 |
| Control | 1.43 ± 0.08 | 1.41 ± 0.09 |

Table of Fig. 5 – Activity of different doses of PBPs from *Hydropuntia cornea* and *Arthrospira platensis* against the *Botrytis cinerea* disease severity of tomato fruits. Data were arcsin transformed. Each value (*n* = 4) is a mean ± SE.

| Treatment mg/mL | *H. cornea* | *A. platensis* |
|---|---|---|
| 0.3 | 45.85 ± 6.07 | 52.43 ± 6.43 |
| 0.6 | 70.28 ± 7.28 | 50.16 ± 7.14 |
| 1.2 | 84.27 ± 7.23 | 56.05 ± 6.73 |
| 2.4 | 118.98 ± 6.12 | 50.35 ± 5.12 |
| 4.8 | 118.50 ± 8.38 | 63.99 ± 2.34 |
| Control | 209.51 ± 8.87 | 197.76 ± 6.82 |

Table of Fig. 7 – Effect of treatment of *Botrytis cinerea* colony portions with different concentrations of PBSs from *Hydropuntia cornea* and *Arthrospira platensis* on colony growth at 3 days after treatment. Data are means values (n = 4) ± SE.

| Treatment mg/mL | *H. cornea* | *A. platensis* |
|---|---|---|
| 0.3 | 45.50 ± 0.61 | 46.63 ± 0.85 |
| 0.6 | 43.50 ± 0.50 | 46.25 ± 0.43 |
| 1.2 | 43.63 ± 0.85 | 47.38 ± 0.55 |
| 2.4 | 41.38 ± 0.24 | 46.50 ± 0.20 |
| 4.8 | 40.25 ± 0.25 | 46.13 ± 0.31 |
| Control | 68.00 ± 0.41 | 69.00 ± 0.82 |

Table of Fig. 8 - Growth of *Botrytis cinerea* colonies on PDA amended with different concentrations of PBSs from *Hydropuntia cornea* and *Arthrospira platensis* at 3 days after inoculation. Data are means values (*n* = 4) ± SE.

| Treatment mg/mL | *H. cornea* | *A. platensis* |
|---|---|---|
| 0.3 | 66.25 ± 0.63 | 65.50 ± 0.65 |
| 0.6 | 66.25 ± 0.85 | 65.25 ± 0.85 |
| 1.2 | 66.00 ± 0.41 | 65.63 ± 0.55 |
| 2.4 | 59.50 ± 0.87 | 65.25 ± 0.63 |
| 4.8 | 57.50 ± 0.87 | 66.00 ± 0.41 |

| | | |
|---|---|---|
| Control | 67.00 ± 0.82 | 69.00 ± 0.41 |

Table of Fig. 9 - Effect of PBPs from *Hydropuntia cornea* and *Arthrospira platensis* on colony forming units (CFUs), and on CFU colony growth of *Botrytis cinerea* at 2 days after spore treatment. Data are mean values ($n$ =4) ± SE.

| Treatment mg/mL | *H. cornea* | *A. platensis* | *H. cornea* | *A. platensis* |
|---|---|---|---|---|
| | CFUs | | CFU colony growth (mm) | |
| 0.3 | 2.00 ± 0.71 | 3.50 ± 0.29 | 1.25 ± 0.25 | 1.50 ± 0.29 |
| 0.6 | 1.00 ± 0.41 | 2.50 ± 0.50 | 0.75 ± 0.25 | 1.25 ± 0.25 |
| 1.2 | 1.00 ± 0.41 | 2.75 ± 0.63 | 0.75 ± 0.25 | 1.25 ± 0.25 |
| 2.4 | 1.00 ± 0.41 | 2.75 ± 0.48 | 0.50 ± 0.20 | 1.25 ± 0.25 |
| 4.8 | 1.25 ± 0.25 | 2.75 ± 0.48 | 0.50 ± 0.20 | 1.25 ± 0.25 |
| Control | 6.50 ± 0.29 | 6.25 ± 0.25 | 2.10 ± 0.4 | 2.90 ± 0.10 |
